# Molecular Characterization, Signaling Activity, and Expression Profiles of a Novel Prolactin Splice Variant (PRL-S) in Zhedong White Geese Across Reproductive States

**DOI:** 10.3390/ijms27167131

**Published:** 2026-08-09

**Authors:** Xiuhua Zhao, Size Wang, Chunwei Wang, Puxuan Zhao, Yue Pan, Chuicheng Zeng, Yuanliang Zhang, Shan Yue, He Huang, Qiuju Wang

**Affiliations:** 1 Animal Husbandry Institute, Heilongjiang Academy of Agricultural Sciences, Harbin 150086, China; yzzxh_007@163.com (X.Z.); zhangyuanliang1218@126.com (Y.Z.); ys924634716@126.com (S.Y.); 2 College of Animal Science and Technology, Northeast Agricultural University, Harbin 150030, China; m15546580001@163.com (S.W.); 19832390209@163.com (P.Z.); 13019655610@163.com (Y.P.); 18236195872@163.com (C.Z.); 3 Heilongjiang Provincial General Station of Animal Husbandry, Harbin 150086, China; snytkjcjg@163.com; 4 College of Animal Science and Veterinary Medicine, Heilongjiang Bayi Agricultural University, Daqing 163319, China

**Keywords:** prolactin, splice variants, broodiness, Zhedong White Geese, egg production

## Abstract

Intense broodiness restricts egg production in Zhedong White Geese, with prolactin (*PRL*) being a key regulator. This study aimed to elucidate the role of *PRL* gene alternative splice variants in regulating broodiness in Zhedong White Geese. A total of 20 Zhedong White Geese at 400 days of age were selected and divided into two groups of 10 based on their physiological states: the laying period and the brooding period. Through bioinformatics and molecular biology approaches, PRL-L and PRL-S were identified and characterized. The results showed that PRL-S lacked a signal peptide and the first 57 amino acids at the N-terminus, leading to the disappearance of its first α-helix structure. Functional validation demonstrated that the recombinant PRL-S protein, prepared using a prokaryotic expression system, possessed physiological activity, including receptor binding and the activation of downstream signaling pathways. Furthermore, Real-time PCR and Western blot analyses revealed that the expression of both splice variants in the hypothalamic–pituitary–ovarian axis exhibited significant spatiotemporal specificity and was closely associated with reproductive states. This study revealed the molecular characteristics, in vitro functional activity, and expression patterns of PRL-S, providing new insights into the regulatory mechanisms of *PRL*.

## 1. Introduction

The Zhedong White Goose is one of the major meat goose breeds in the coastal regions of southeastern China. Due to its fast growth rate, high meat quality, and strong tolerance to roughage, this breed plays an important role in modern waterfowl production [1,2]. However, this breed exhibits notable deficiencies in reproductive performance, primarily characterized by low egg production and intense broodiness [3,4]. Broodiness is an instinctual reproductive behavior formed through long-term evolution in poultry, physiologically manifested by the cessation of egg-laying, nest-building and incubation behaviors, and significant ovarian regression [2,5]. In modern intensive farming systems, artificial incubation technology has efficiently replaced natural incubation [6]. Consequently, natural broodiness in geese not only leads to a waste of incubation resources but also significantly prolongs the inter-clutch interval, and is therefore considered one of the major factors restricting the improvement of their annual egg production rate [7]. Therefore, an in-depth elucidation of the physiological regulatory mechanisms underlying broodiness in Zhedong White Geese, and the subsequent suppression of this trait through genetic breeding, holds great scientific significance and practical value for shortening the non-laying period, unlocking reproductive potential, and enhancing the economic benefits of the industry.

Prolactin (*PRL*) is widely recognized as a key endocrine factor that induces and maintains broodiness in poultry [8]. As a single-gene-encoded polypeptide hormone secreted by the pituitary gland, *PRL* has a molecular weight of approximately 23 kDa, and its spatial structural stability relies on three disulfide bonds formed by six conserved cysteine residues within the molecule [9]. The regulatory effect of *PRL* on the reproductive axis is concentration- and tissue-dependent. Studies have shown that low to moderate concentrations of *PRL* may promote follicular development, whereas high concentrations are associated with the induction of broodiness and follicular atresia [10]. The plasma concentrations of *PRL* in poultry exhibit distinct cyclical fluctuations, maintaining low levels during the laying period and surging significantly towards the end of the laying cycle and during the broody period [11]. It is noteworthy that the expression products of the *PRL* gene are not singular; including ‘transcript variants’ produced via alternative splicing, and ‘protein isoforms or modified forms’ arising from proteolytic cleavage and post-translational modifications [12]. The spatiotemporal differential distribution of these variants may exert more refined regulatory effects on cell proliferation, differentiation, and specific reproductive physiological states, providing a novel molecular perspective for explaining individual differences in poultry reproductive performance.

Although the functions of *PRL* splice variants in mammals have been extensively studied, it remains unclear whether the *PRL* gene undergoes alternative splicing in waterfowl (such as geese), what its specific molecular characteristics are, and the precise mechanisms by which these splice variants regulate broodiness [13,14,15]. Therefore, this study hypothesized that the *PRL* gene in Zhedong White Geese generates multiple transcript variants via alternative splicing, which exhibit structural and functional differences and collectively regulate broodiness. This study not only elucidated the molecular characteristics of *PRL* in Zhedong White Geese from the perspective of gene structural diversity but also provided solid experimental evidence for a deeper understanding of the specific molecular mechanisms by which *PRL* and its splice variants regulated broodiness in poultry. Moreover, it offered a potential future application that still required validation for future molecular breeding programs aimed at developing new lines of Zhedong White Geese with reduced broodiness and higher egg production.

## 2. Results

### 2.1. Identification and Validation of PRL Alternative Splice Variants

**Figure 1:**
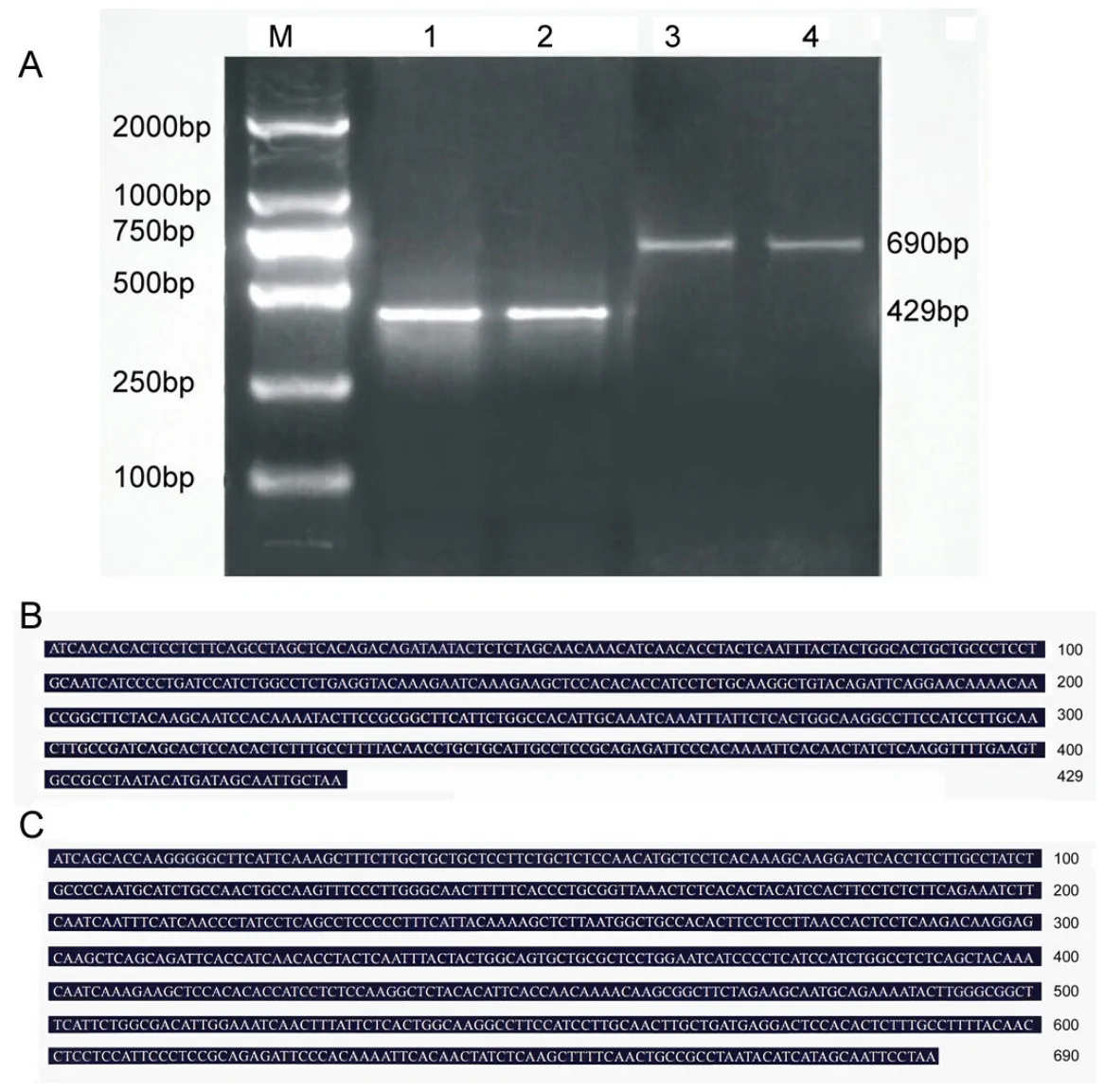
Validation of *PRL* alternative splice variants. (**A**) PCR validation of *PRL* alternative splice variants; (**B**) Sequencing results of the PCR amplified band for PRL-S; (**C**) Sequencing results of the PCR amplified band for PRL-L. M: Marker (2000 bp, 1000 bp, 750 bp, 500 bp, 250 bp, 100 bp); 1 and 2: PRL-S; 3 and 4: PRL-L.

Based on reference genome sequencing and sequence analysis, primers were designed to amplify the full-length sequences of the *PRL* alternative splice variants for transcriptomic validation. The PCR products were analyzed via 2% agarose gel electrophoresis, which revealed two distinct bands, a longer band corresponding to PRL-L, matching the full-length CDS of the *PRL* gene, and a shorter band corresponding to PRL-S (Figure 1A). Sanger sequencing of the PCR products was consistent with the transcriptome sequencing results. The full-length CDS of PRL-S was 429 bp, encoding a 142-amino acid protein. Bioinformatic analysis revealed that this protein lacked a typical signal peptide sequence; therefore, its 142 amino acids constituted the length of the mature peptide, with a theoretical molecular weight of approximately 17 kDa (Figure 1B). The full-length CDS of PRL-L was 690 bp, encoding a precursor protein of 229 amino acids. Its N-terminus contained a 30-amino acid signal peptide. After the removal of the signal peptide, the mature PRL-L protein consisted of 199 amino acids, with a theoretical molecular weight of approximately 23 kDa (Figure 1C). The experiment was independently repeated three times, and the image shown was representative.

### 2.2. Analysis of Sequence Characteristics and Gene Structure of PRL Alternative Splice Variants

**Figure 2:**
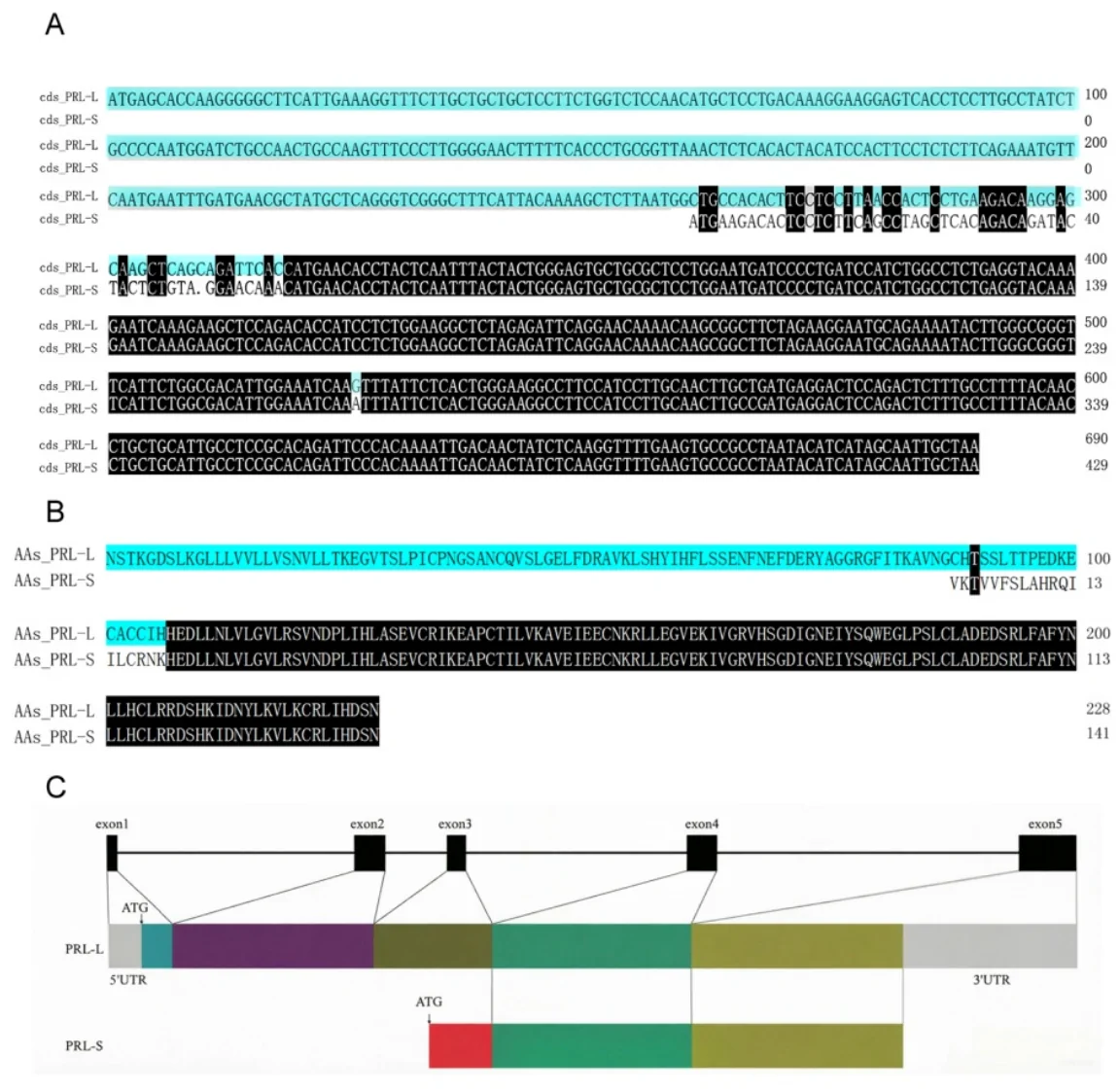
Alignment of gene and protein sequences and gene structure of *PRL* alternative splice variants. (**A**) Alignment of CDS sequences; (**B**) Alignment of polypeptide sequences; (**C**) Schematic diagram of the exon sequences of PRL-S and PRL-L.

Alignment of the mRNA sequences of the *PRL* alternative splice variants and their corresponding polypeptide sequences revealed that PRL-S lacked 261 nucleotides at the 5′ end compared to PRL-L, and the first 57 nucleotides at the 5′ end of PRL-S did not perfectly match those of PRL-L. Within the remaining 367 nucleotides of PRL-S, an A>G mutation was identified at the 265th nucleotide (corresponding to the 526th nucleotide in PRL-L), and a T>C mutation was found at the 306th nucleotide (corresponding to the 567th nucleotide in PRL-L) (Figure 2A). These substitutions represented sequence differences between the PRL-S and PRL-L transcript variants, rather than inter-individual single nucleotide polymorphisms or sequencing errors. Alignment of the amino acid sequences showed that PRL-S lacked 87 amino acids compared to the PRL-L precursor protein (including the 30-amino-acid signal peptide sequence), and the first 19 amino acids at the N-terminus of PRL-S did not perfectly match those of PRL-L. Due to the A>G mutation at the 265th nucleotide in the PRL-S sequence, a Val>Ile substitution was observed at the 89th amino acid position (corresponding to the 146th amino acid in PRL-L) (Figure 2B). Gene structure analysis indicated that the *PRL* gene comprised five exons and four introns. Sequence alignment was performed using SnapGene software 8.2.2. The coding sequence of PRL-L consists of all five exons, whereas the coding sequence of PRL-S was composed of a divergent sequence segment followed by exons 4 and 5 (Figure 2C). The unique sequence preceding exons 4 and 5 in PRL-S (the red region in Figure 2C) originated from a cryptic 5′ splice site located within the third exon of PRL-L. During the splicing of PRL-S, most of exons 1, 2, and 3 of PRL-L were skipped; splicing initiated directly from this cryptic site and joins to exons 4 and 5, thereby generating this unique N-terminal sequence.

**Figure 3:**
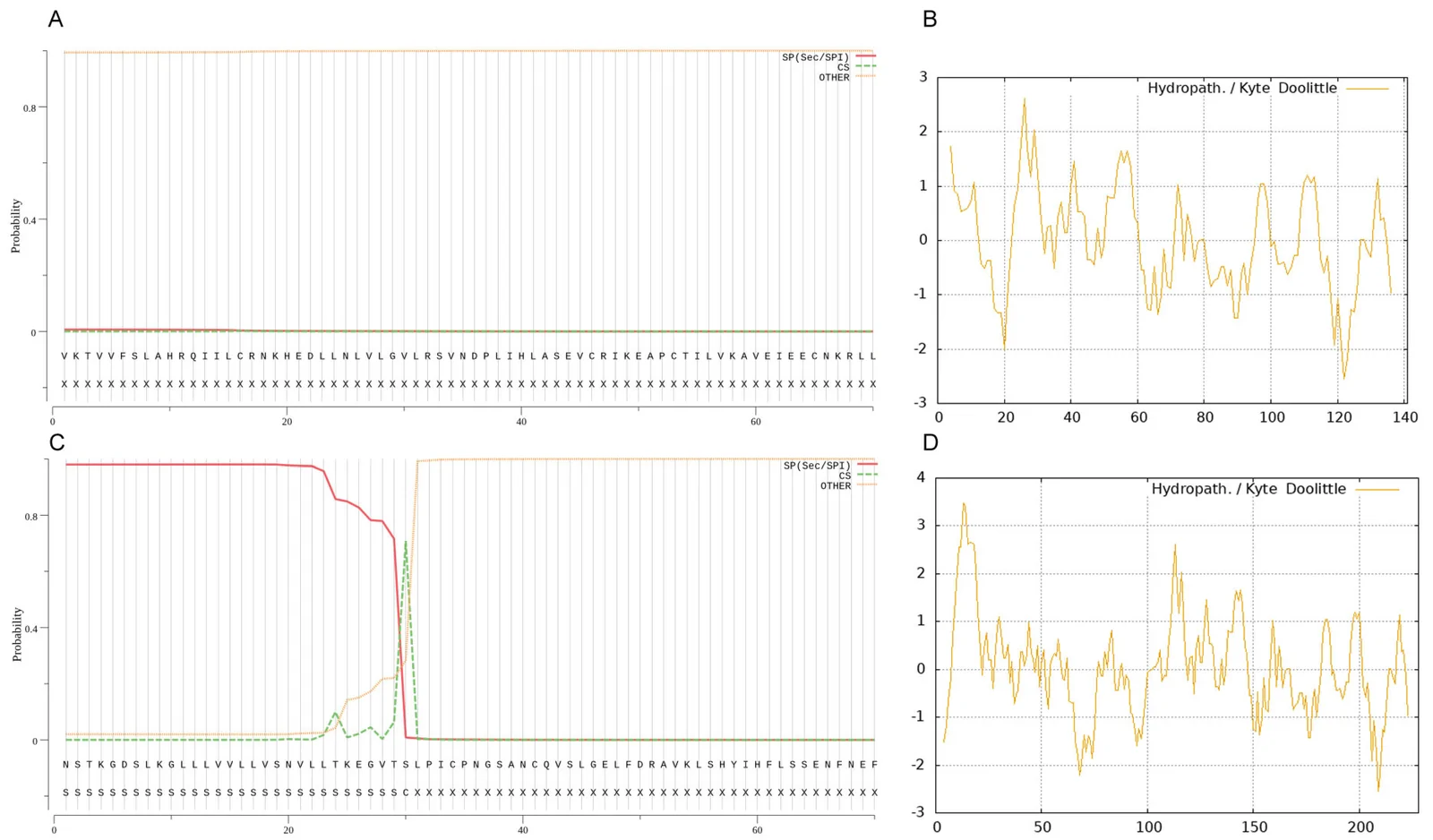
Signal peptide prediction and hydrophilicity/hydrophobicity analysis of *PRL* alternative splice variant proteins. (**A**) Signal peptide prediction for PRL-S; (**B**) Hydrophilicity/hydrophobicity analysis of the PRL-S polypeptide. The box indicates the 19-amino-acid region at the N-terminus of PRL-S that does not perfectly match the mature peptide of PRL-L. The x-axis represents the sequence position, and the y-axis represents the amino acid scale value (values > 0 indicate hydrophobicity, and values < 0 indicate hydrophilicity); (**C**) Signal peptide prediction for PRL-L; (**D**) Hydrophilicity/hydrophobicity analysis of the PRL-L polypeptide. SP: the probability of a secretory pathway signal peptide; CS: the probability of the signal peptide cleavage site; OTHER: the probability of a non-signal peptide region; S: signal peptide, X: non-signal peptide, C: cleavage site.

Signal peptide prediction revealed that the first 30 amino acids of the PRL-L polypeptide constituted a signal peptide, whereas no signal peptide sequence existed in the PRL-S polypeptide (Figure 3A,C). Hydrophilicity/hydrophobicity analysis indicated that both proteins were primarily composed of hydrophilic amino acids. The 19-amino-acid region at the N-terminus of PRL-S that did not perfectly match the mature peptide of PRL-L exhibited significant differences in hydrophilicity/hydrophobicity (Figure 3B,D and Table 1).

### 2.3. Analysis of Secondary and Tertiary Structures of PRL Alternative Splice Variant Proteins

**Figure 4:**
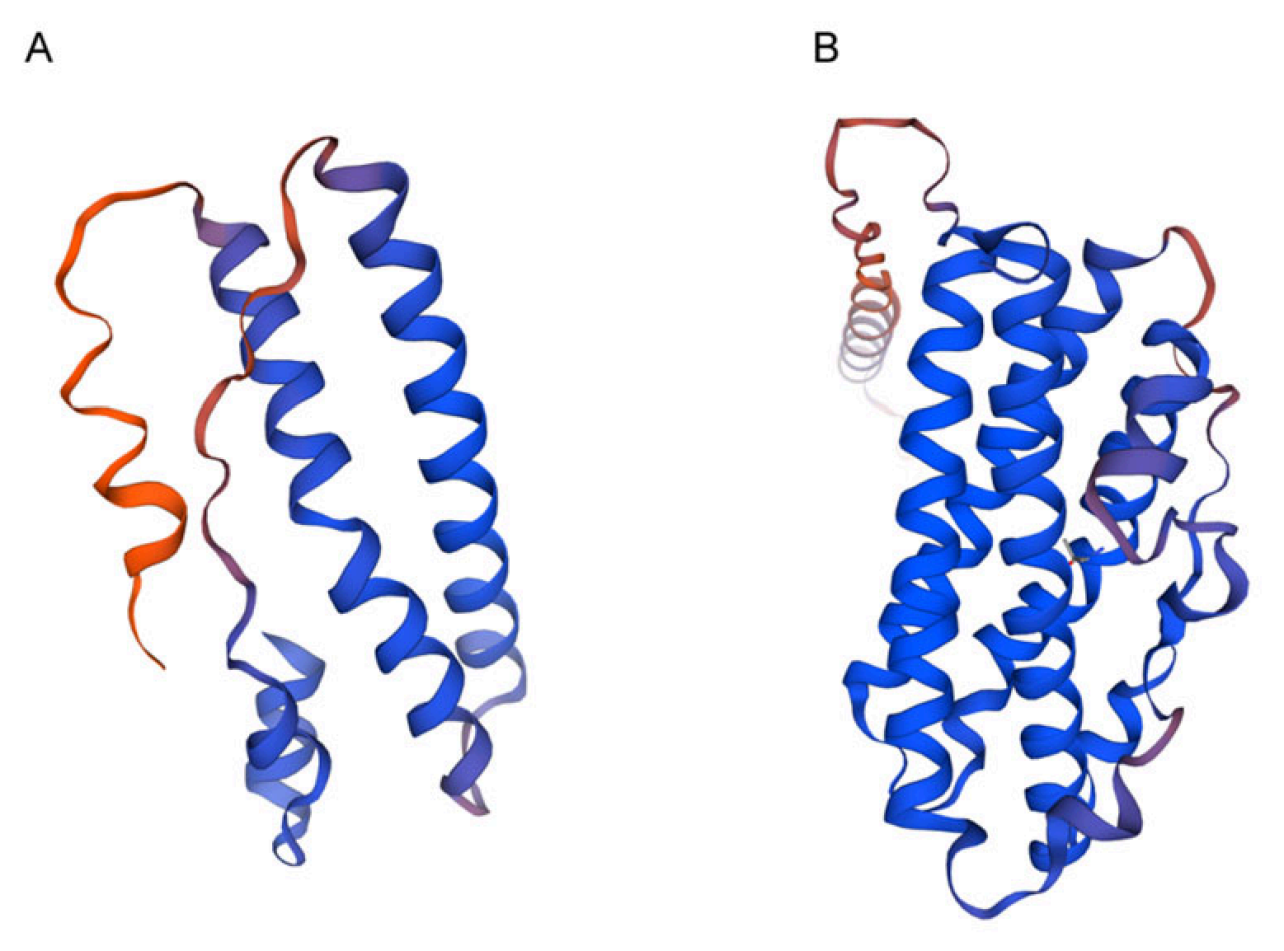
Tertiary structures of *PRL* alternative splice variants. (**A**) PRL-S, (**B**) PRL-L.

As shown in Table 2, the proportions of α-helix, extended strand, β-turn, and random coil in the 142 amino acids of PRL-S were 67.61%, 1.41%, 2.11%, and 28.87%, respectively. In the 199 amino acids of PRL-L, these proportions were 61.81%, 1.01%, 2.51%, and 34.67%, respectively. The tertiary structure models of the PRL-S and PRL-L proteins were constructed using the Swiss-Model online server (Figure 4). For the PRL-S protein, the GMQE value was 0.74 and the GMEAN value was 0.74; for the PRL-L protein, the GMQE value was 0.79 and the GMEAN value was −1.64. A GMEAN value greater than −4.0 indicates high model quality. As illustrated in Figure 4, the three-dimensional structures of the two PRL proteins were generally similar. Based on the N-terminal sequence alignment, the divergent sequence between the two proteins was located within the first 76 amino acids of the PRL-L N-terminus, comprising a deletion of 57 amino acids and mutations in 19 amino acids. The mature PRL-L protein contained four intact α-helices, whereas PRL-S lacked the first α-helix. These structural differences may potentially lead to changes in their molecular characteristics and functions.

### 2.4. Expression and Analysis of PRL Fusion Proteins

**Figure 5:**
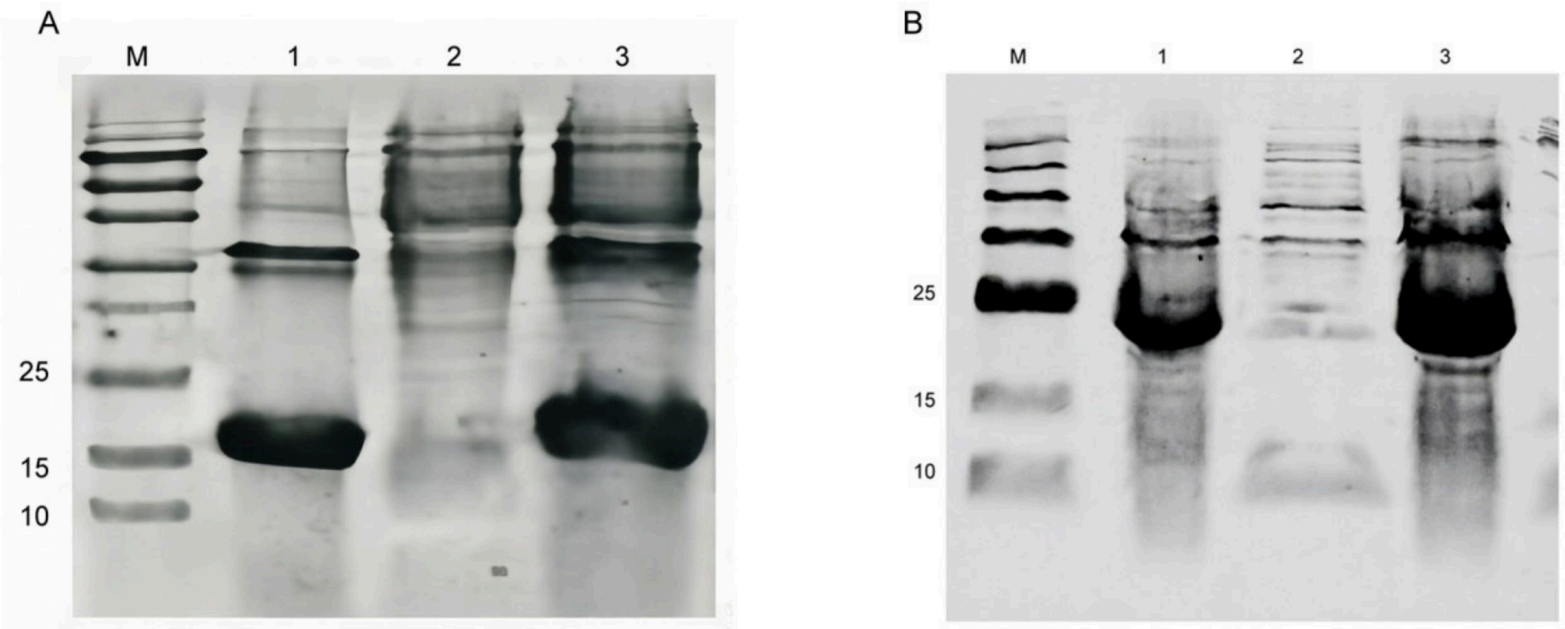
Prokaryotic expression of recombinant PRL-S and PRL-L proteins. (**A**) PRL-S; (**B**) PRL-L; M: Protein marker; 1: Induced expression; 2: Supernatant after induced expression; 3: Pellet after induced expression.

The molecular weights of the PRL-S and PRL-L fusion proteins were approximately 17 kDa and 23 kDa, respectively, which were consistent with the expected sizes. Both the PRL-S and PRL-L fusion proteins were detected in the pellet of the bacterial lysate, indicating that they were expressed in the form of inclusion bodies (Figure 5).

### 2.5. Purification and Refolding of PRL Fusion Proteins

**Figure 6:**
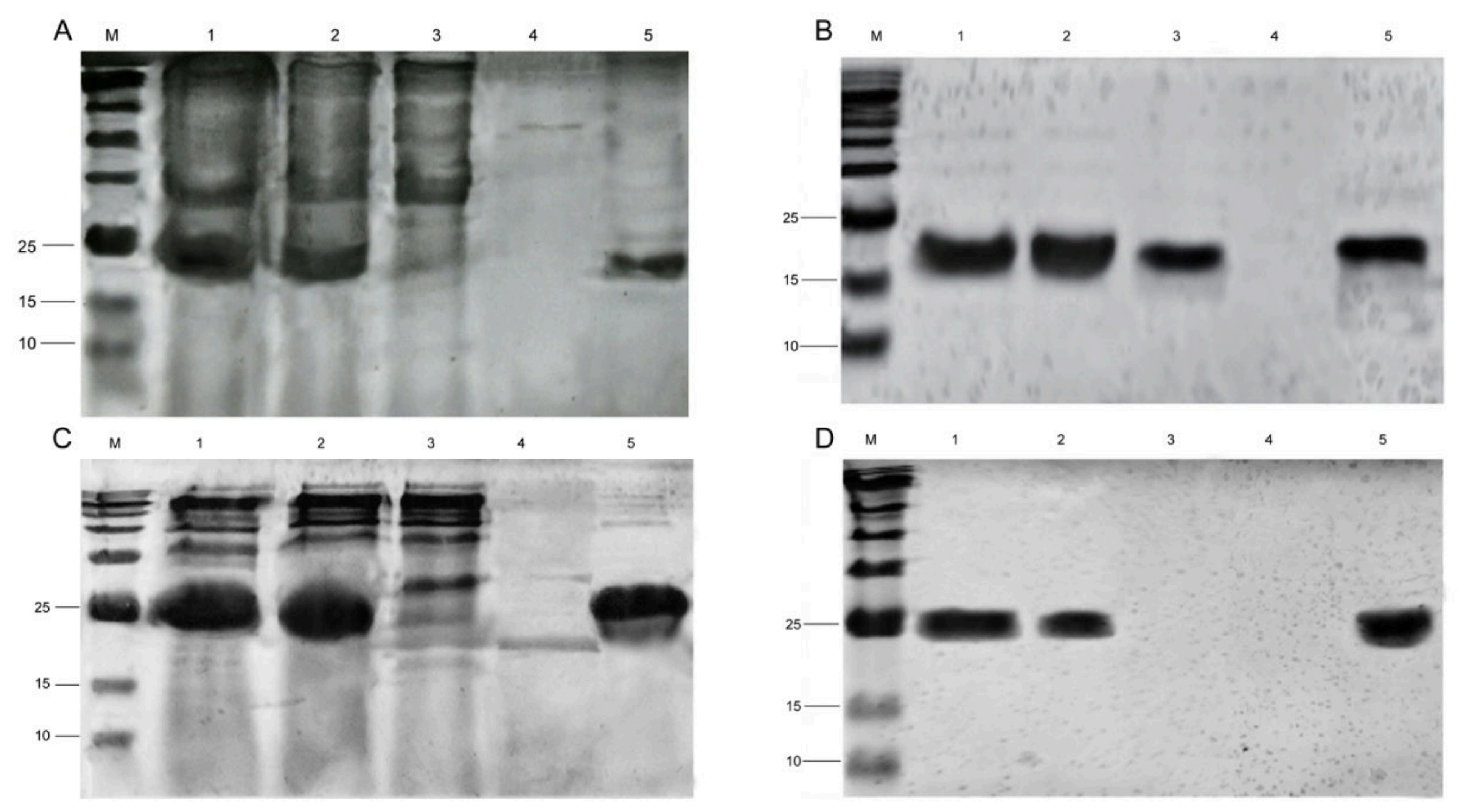
Purification and refolding of recombinant PRL-S and PRL-L proteins. (**A**) Purification of recombinant PRL-S protein. M: Protein Marker; 1. Inclusion body solubilization buffer; 2. Supernatant of inclusion body solubilization buffer; 3. Purification flow-through; 4. Purification wash buffer; 5. Purification elution. (**B**) Refolding of recombinant PRL-S protein. 1. Recombinant protein solution (in Buffer A); 2. Supernatant of recombinant protein solution (in Buffer A); 3. Refolding flow-through; 4. Refolding wash buffer; 5. Refolding elution. (**C**) Purification of recombinant PRL-L protein. 1. Inclusion body solubilization buffer; 2. Supernatant of inclusion body solubilization buffer; 3. Purification flow-through; 4. Purification wash buffer; 5. Purification elution. (**D**) Refolding of recombinant PRL-L protein. 1. Recombinant protein solution (in Buffer A); 2. Supernatant of recombinant protein solution (in Buffer A); 3. Refolding flow-through; 4. Refolding wash buffer; 5. Refolding elution.

The molecular weight and band width of the fusion protein in the bacterial lysate and the inclusion body solubilization buffer were consistent, indicating that the fusion proteins were completely dissolved in the Binding Buffer. No fusion protein bands were detected in the flow-through fraction, demonstrating that all the fusion proteins successfully bound to the Ni magnetic beads. Only the fusion protein bands, without any other protein bands, were observed in the elution fraction, confirming the successful purification of the fusion proteins (Figure 6). In the refolding solution, the fusion protein bands were consistent with those from the other steps and showed no other protein bands, indicating that the fusion proteins were soluble and non-degraded after refolding. Protein concentration assays showed that the concentrations of the PRL-L and PRL-S fusion protein solutions were 0.268 mg/mL and 0.294 mg/mL, respectively. The purity of both recombinant proteins was greater than 95%.

### 2.6. Co-IP Verification of the Physiological Activity of Recombinant PRL Proteins

**Figure 7:**
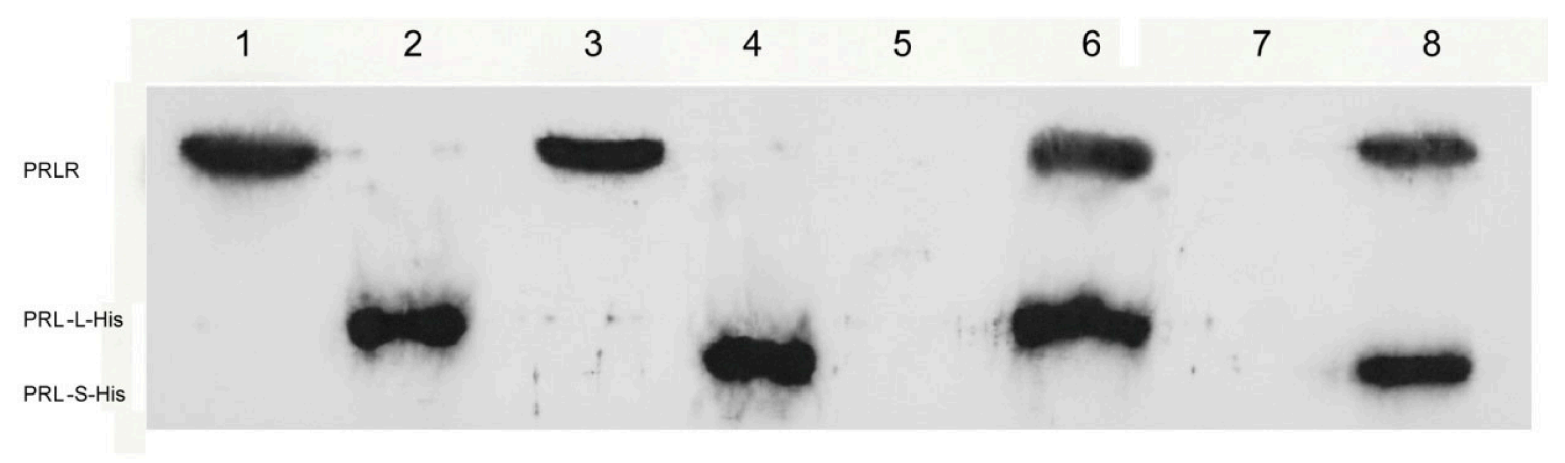
Co-IP verification of the interaction between recombinant PRL-S and PRL-L proteins. 1 and 3: SYF Lysate, 2: Recombinant PRL-L Solution, 4: Recombinant PRL-S Solution, 5 and 7: IgG, 6 and 8: anti-*PRLR*.

The PRL-His recombinant protein solution contained the PRL-His recombinant protein but lacked the *PRLR*. Conversely, the cell lysate contained *PRLR* but lacked the PRL-His fusion protein. In the Co-IP elution fraction, both the PRL-His recombinant protein and *PRLR* were detected. However, neither protein was present in the control group where rabbit IgG was used instead of the anti-*PRLR* antibody. These results indicated that the denatured and refolded PRL-His recombinant protein can successfully bind to *PRLR*, retaining a functional receptor-binding conformation and exhibiting physiological activity. Furthermore, the mutation in the first α-helix of the PRL protein’s three-dimensional structure did not affect the interaction between *PRL* and *PRLR* (Figure 7).

### 2.7. PRL-S Proteins Binding to PRLR Activate Downstream Pathways

**Figure 8:**
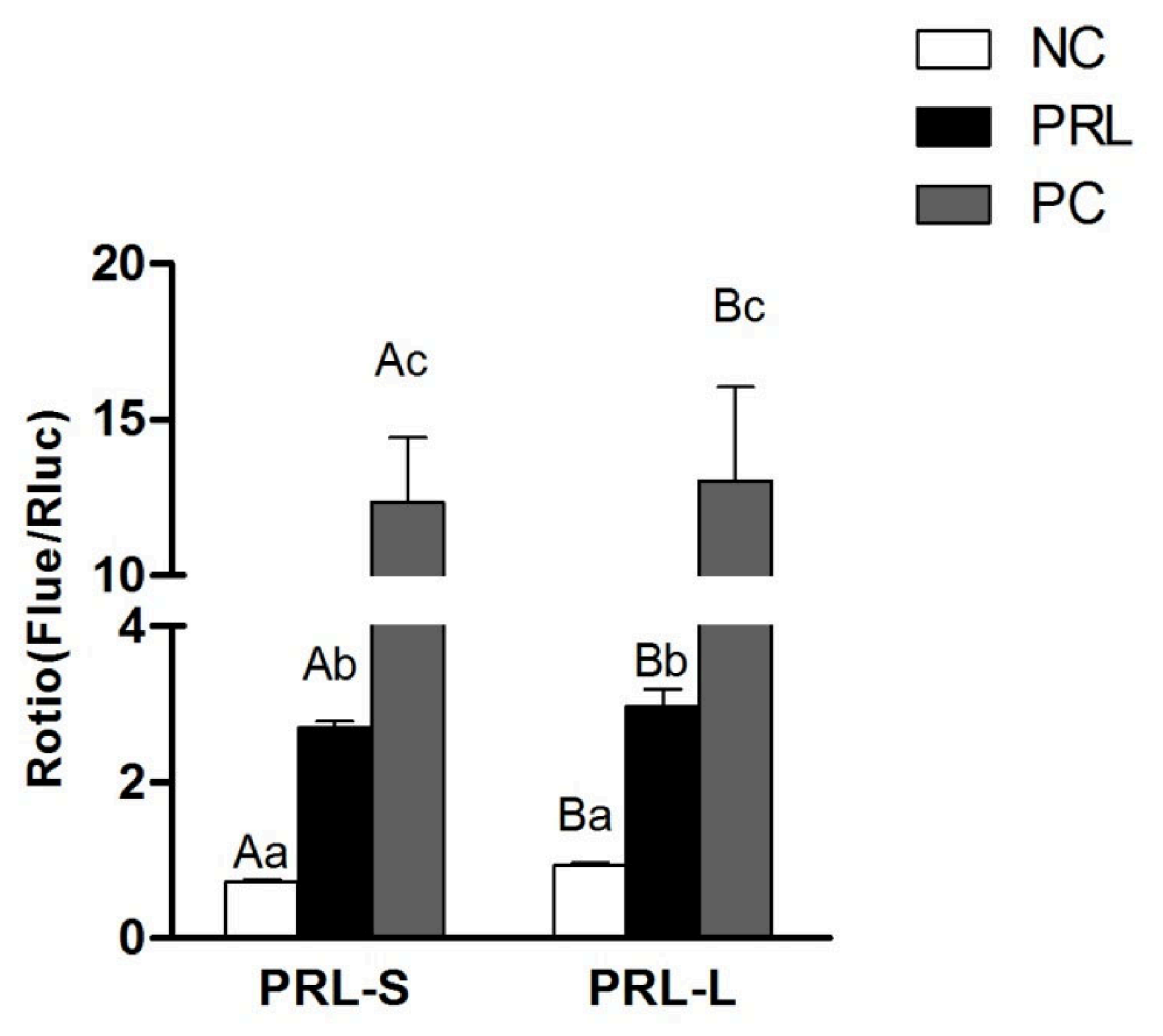
Activity assessment of *PRL* using a dual-luciferase reporter system. NC: Negative Control, *PRL*: Recombinant PRL protein treatment, PC: Positive Control. HEK293T cells transfected with pGL3-control, pGL3-5× (*STAT5*), and pRL-TK vectors served as the positive control. HEK293T cells transfected with pRL-TK plasmid and pGL3-5× (*STAT5*) vector served as the negative control. Bar graphs represent mean ± SD. In the same group, the bars with different capital letters indicate significant differences between different periods (*p* < 0.05), while the bars with the same letters are not significant different (*p* > 0.05). At the same gene, bars with different lowercase letters represent significant differences between different genes (*p* < 0.05); bars with the same letters are not significant different (*p* > 0.05). *n* = 3.

As assessed by the dual-luciferase reporter system and shown in Figure 8, the firefly luciferase activity in Zhedong White Goose *PRLR*-transfected HEK293T cells treated with recombinant PRL proteins was significantly higher than that in untreated cells. These results indicated that the recombinant PRL proteins can activate intracellular signal transduction pathways via *PRLR*, thereby inducing cellular responses. The recombinant PRL-S protein exhibited comparable potential to PRL-L in activating the *JAK-STAT* signaling pathway.

### 2.8. Expression of PRL Alternative Splice Variants in the Hypothalamus, Pituitary, and Ovary

**Figure 9:**
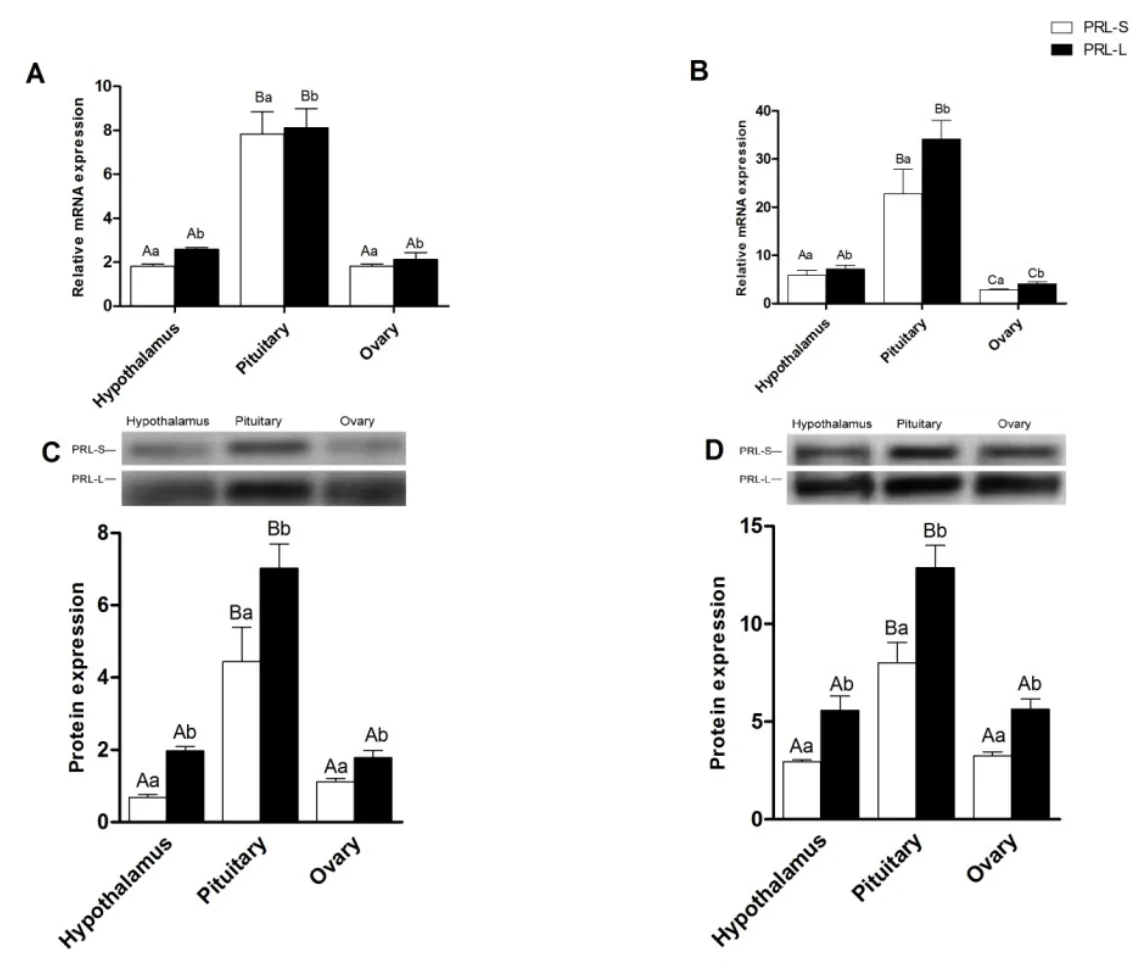
Relative mRNA and protein expression of PRL-S and PRL-L in the hypothalamus, pituitary, and ovary during the laying and broody period. (**A**) The mRNA expression level of PRL-S and PRL-L in the hypothalamus, pituitary and ovary during the laying period, (**B**) The mRNA expression level of PRL-S and PRL-L in the hypothalamus, pituitary and ovary during the broody period. (**C**) The protein expression level of PRL-S and PRL-L in the hypothalamus, pituitary and ovary during laying period, (**D**) The protein expression level of PRL-S and PRL-L in the hypothalamus, pituitary and ovary during the broody period. Bar graphs represent mean ± SD (*n* = 10). At the same gene, the bars with different capital letters indicate significant differences between different periods (*p* < 0.05), while the bars with the same letters are not significant different (*p* > 0.05). In the same period, bars with different lowercase letters represent significant differences between different genes (*p* < 0.05); bars with the same letters are not significant different (*p* > 0.05).

The mRNA and protein expression levels of PRL-S and PRL-L in the hypothalamus, pituitary, and ovary of geese were examined using qRT-PCR and Western blot assays. The results were presented in Figure 9. The *PRL* alternative splice variants were expressed in all examined tissues during both the laying period and the broodiness period in geese. Furthermore, within the same tissue, the expression level of PRL-L was significantly higher than that of PRL-S (*p* < 0.05), and the expression levels of both were significantly higher during the broody period than during the laying period (*p* < 0.05). Additionally, they exhibited the highest expression in the pituitary during the two periods (*p* < 0.05). Interestingly, the protein expression profile of PRL-S and PRL-L was highly consistent with the mRNA expression profile.

### 2.9. Expression of PRL Alternative Splice Variants in Follicles at Various Developmental Stages

**Figure 10:**
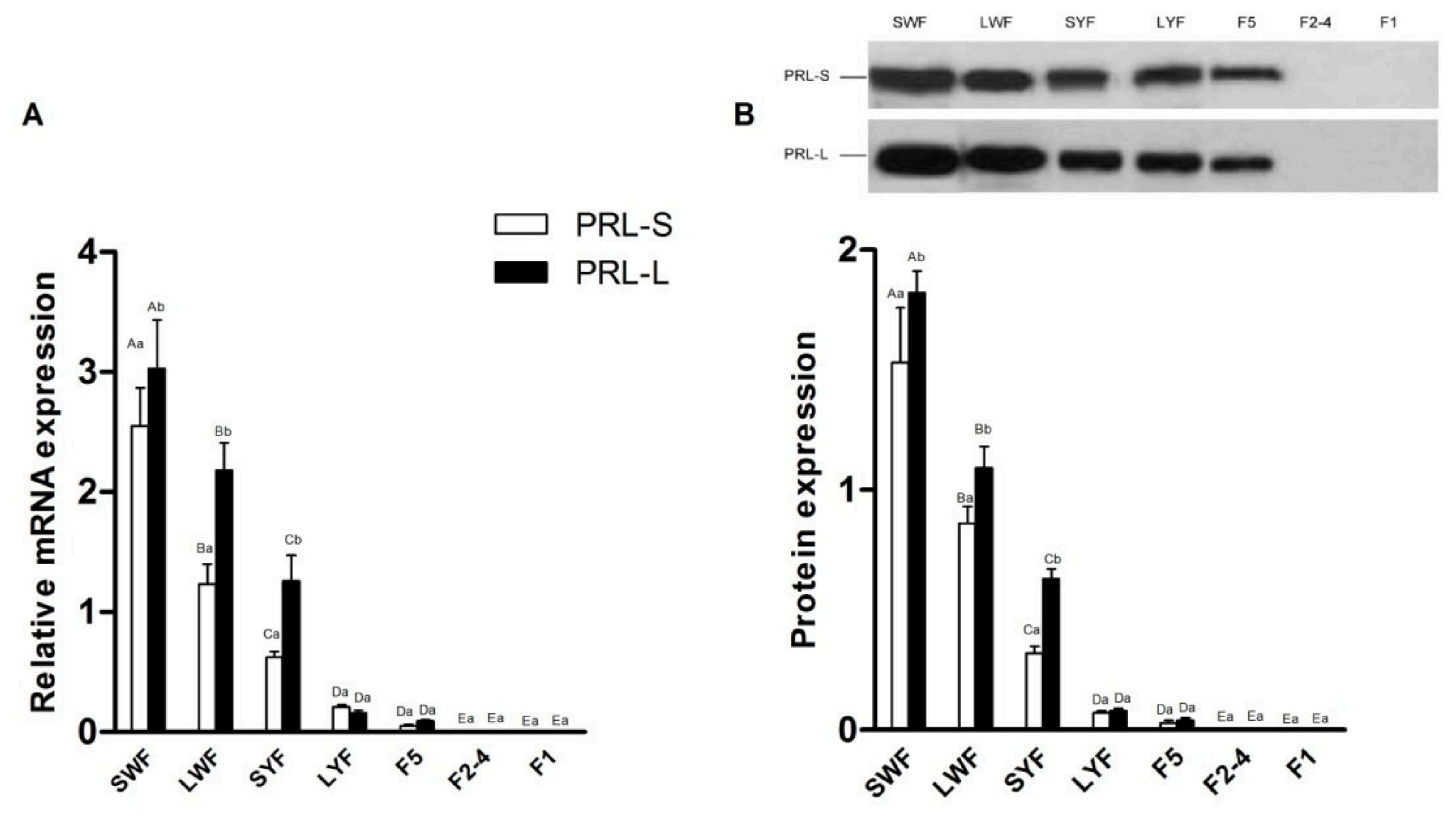
PRL-S and PRL-L expression in follicles of Zhedong White Geese at various stages. (**A**) The mRNA expression level of PRL-S and PRL-L in follicles; (**B**) The protein expression level of PRL-S and PRL-L in follicles. Bar graphs represent mean ± SD (*n* = 10). In the same gene, the bars with different capital letters indicate significant differences among different follicles (*p* < 0.05), while the bars with the same letters are not significant different (*p* > 0.05). In the same follicle, bars with different lowercase letters represent significant differences between different genes (*p* < 0.05); bars with the same letters are not significant different (*p* > 0.05).

The expression patterns of *PRL* alternative splice variants in follicles at different developmental stages were illustrated in Figure 10. The mRNA expression levels of both PRL-S and PRL-L exhibited a significant downward trend as follicular development progressed. Expression was highest during the early stages of small white follicles (SWF) and large white follicles (LWF). Subsequently, expression gradually declined to extremely low levels in small yellow follicles (SYF), large yellow follicles (LYF), and yellow follicles of all hierarchical grades (F5 to F1). In SWF, the expression levels of both PRL-S and PRL-L reached their peak, with the relative expression level of PRL-L being significantly higher than that of PRL-S (*p* < 0.05). As follicles enlarged into the LWF stage, the expression levels of both PRL-S and PRL-L decreased significantly compared to the SWF stage (*p* < 0.05). However, both maintained relatively high levels, and the expression of PRL-L remained significantly higher than that of PRL-S (*p* < 0.05). Upon entering the SYF stage, *PRL* expression decreased significantly further. During this stage, the expression level of PRL-L remained significantly higher than that of PRL-S (*p* < 0.05). In LYF, *PRL* expression continued to decline significantly. During this stage, the difference in expression between PRL-S and PRL-L was no longer statistically significant (*p* > 0.05). From F5 to F1, the mRNA expression levels of both PRL-S and PRL-L were maintained at extremely low levels, with no significant differences observed. This indicated that during the phases of rapid follicular growth and maturation in preparation for ovulation, local transcriptional activity of the *PRL* gene within the follicle was strongly suppressed.

## 3. Discussion

*PRL* is one of the crucial reproductive hormones in avian species, playing a vital role in regulating seasonal reproduction [16]. As birds enter the breeding season, *PRL* concentrations gradually rise, reaching their peak during the late reproductive stage [17]. Low to moderate concentrations of *PRL* promote ovarian follicular development and egg production in birds, whereas high concentrations induce follicular atresia and broodiness, thereby inhibiting egg-laying [18]. Alternative mRNA splicing is one of the mechanisms responsible for generating *PRL* variants [19]. In this study, we identified and characterized a novel alternative splice variant of *PRL* in Zhedong White Geese, designated as PRL-S. This significant difference in sequence length directly resulted in the truncated nature of its encoded product, constituting the most fundamental structural feature of PRL-S. Alignment analysis of the amino acid sequences of PRL-S and PRL-L revealed fundamental variations in the N-terminal domain. This drastic alteration in the N-terminal sequence, combined with bioinformatics predictions of protein secondary structure, led to a major remodeling of the spatial conformation of PRL-S. The α-helix was a critical secondary structural element for maintaining the specific three-dimensional conformation and function of proteins, its absence may profoundly affect the overall folding, stability, and interaction interfaces with receptors or other molecules of PRL-S. The instability index is a computational prediction based on the primary sequence of a protein, and a value greater than 40 suggests that the protein may be unstable in vitro. However, this does not necessarily reflect its actual stability in vivo, as complex intracellular environmental factors, such as chaperones and post-translational modifications, can also affect protein stability. Furthermore, our experimental results (successful expression, purification, and acquisition of an active protein) confirmed that it can exist stably under specific conditions.

Further analysis of the PRL-S protein sequence revealed that this splice variant completely lacked the signal peptide sequence essential for classical secretory proteins. In avian species, the mature *PRL* peptide was preceded by a 30-amino acid signal peptide that directs the newly synthesized PRL protein into the endoplasmic reticulum-Golgi secretory pathway, ultimately secreting it into the extracellular space as a hormone to act on target organs via the bloodstream [20]. The absence of a signal peptide in PRL-S implied that it cannot be released into the extracellular environment through the classical secretory pathway. This structural feature challenged the traditional notion that *PRL* must function as a secretory hormone and introduces new hypotheses regarding the mechanism of action of PRL-S [21]. Based on this, PRL-S may be released via a non-classical secretory mechanism or, more likely, primarily functions as an intracellular signaling molecule as part of intracellular signal transduction pathways. This reconstructed functional trajectory suggested that PRL-S may play a biological role entirely distinct from that of the classical endocrine/paracrine PRL-L. Furthermore, the in vitro addition of recombinant proteins in this study was intended solely to verify their ‘ligand potential’, rather than to definitively establish their exclusive physiological secretion mechanism. Although PRL-S exhibited critical deletions and variations in the N-terminal domain, experimental results demonstrated that it still retained the ability to bind to the *PRLR*. We hypothesize that PRL-S may not rely on the classical N-terminal binding domain but instead utilized its unique N-terminal 19-amino acid sequence or other conserved C-terminal regions to form an alternative, non-classical binding mode with *PRLR*. This novel binding interface may induce a different dimerization conformation of *PRLR*, thereby affecting the recruitment efficiency and specificity of downstream signaling molecules, ultimately resulting in differences in the strength, duration, or preference of the signaling pathways activated compared to PRL-L. Therefore, the structural variation in PRL-S did not simply lead to a loss of function; rather, it likely signified a functional switch or isoform specificity, reflecting the evolutionary strategy of achieving functional diversification through alternative splicing.

To verify whether PRL-S retained the core biological function of binding to the *PRLR*, a Co-IP assay was employed. The experimental results demonstrated that the recombinant PRL-S protein can specifically bind to *PRLR*. This finding held significant biological implications. Despite the deletion of 57 N-terminal amino acids in PRL-S mature peptide, including the critical receptor-binding region (amino acids 57–90) in PRL-L, its ability to still bind *PRLR* challenged the traditional understanding of *PRL* functional domains. This result suggested that PRL-S may form an alternative receptor-binding interface through its unique N-terminal 19-amino acid sequence or other conserved regions, thereby maintaining its interaction capability with *PRLR*. This “non-classical” binding mode may indicate that PRL-S possessed signal transduction characteristics or functions distinct from those of the PRL-L. However, there were limitations associated with the refolding of recombinant proteins, and the in vitro results need to be further validated in an in vivo context. Upon binding to *PRLR*, the classical signal transduction pathway involved the activation of the *JAK-STAT* pathway [22]. The experimental data showed an upward trend in reporter gene activity following PRL-S treatment. This result supported the possibility that PRL-S can activate the *JAK-STAT* signaling pathway. Although its activation efficiency may differ from that of PRL-L, this assay provided critical evidence for PRL-S functioning as a functional ligand. Study in rats provided direct mechanistic evidence that PRL-S activated the *JAK-STAT* pathway. This research found that during lactogenesis, mammary tissue responded to PRL-S by activating Janus kinase 2 (*JAK2*) and Signal Transducer and Activator of Transcription 5 (*STAT5*), directly demonstrating that PRL-S mediated signal transduction through the *PRLR-JAK2-STAT5* pathway [23]. Mangoura et al. found that *PRL* activated *JAK-STAT* signaling pathways to induce proliferation while promoting differentiation in embryonic astrocytes [24]. These findings collectively reinforced the notion that PRL-S functions via similar mechanisms. Due to experimental limitations, this study primarily relied on the response of HEK293T cells transfected with the *PRLR* plasmid to evaluate function. Future studies will include empty vector transfection controls to further validate receptor specificity. Notably, although the *JAK-STAT* pathway is highly conserved, the interaction between goose *PRLR* and human kinases may exhibit species-specific differences, necessitating further validation in homologous cell lines.

The *PRL* alternative splice variant, PRL-S, identified in Zhedong White Geese in this study, exhibited a highly spatiotemporal-specific expression pattern. Experiments demonstrated that PRL-S expression was highest in Zhedong White Geese reared under short photoperiods, specifically within SWF. *PRL* expression in follicles declined to extremely low levels as development progresses. This may suggest that during the mature follicle stage, *PRL* was primarily secreted by the pituitary (endocrine action) rather than synthesized locally (paracrine/autocrine). Alternatively, local *PRL* may play a role in early follicular development and be suppressed in later stages to prevent atresia. This expression pattern was closely associated with specific reproductive physiological states. In the avian reproductive cycle, the developmental status of SWFs was a critical node determining subsequent egg-laying performance [25]. The presence of PRL-S under short photoperiods and in SWF follicles strongly suggested its potential involvement in regulating the critical transition from follicular development to ovulation. Linking this expression pattern to the favorable reproductive phenotypes exhibited by Zhedong White Geese under short photoperiods, it can be inferred that PRL-S was not a non-functional byproduct, but rather a refined regulatory molecule evolved by the organism to adapt to specific light environments and optimize reproductive input and output. Its specific expression pattern constituted the primary spatiotemporal evidence for exploring its physiological functions.

As the most important environmental factor, photoperiod plays a central role in regulating seasonal reproduction in birds, with *PRL* serving as a key effector molecule in this regulatory pathway [26]. Multiple studies across different goose breeds had confirmed the precise regulation of *PRL* secretion by photoperiod. Liu et al. found that Magang geese had shown distinct seasonal variations in their reproductive activities. Under natural conditions, during the non-breeding season (April to July), plasma PRL concentrations rose to 20–30 ng/mL while luteinizing hormone (LH) concentrations dropped below 1 ng/mL, resulting in molting and the cessation of egg-laying. Upon entering the breeding season (August to March of the following year), plasma PRL concentrations remained at a low level of approximately 10 ng/mL, whereas LH concentrations increased to 1–2 ng/mL, leading to the resumption of egg-laying [27]. Artificial photoperiod control experiments had further validated this causal relationship, prolonged light exposure simulated a non-breeding state, inducing elevated plasma PRL and decreased LH, which caused hens to enter a resting period and molt. Conversely, shortened photoperiods reduced PRL *co*ncentrations (to 10 ng/mL) and promoted LH secretion (2–3 ng/mL), thereby inducing hens to enter the laying period [28]. These results clearly demonstrated that photoperiod coordinates seasonal reproductive activities in Magang geese primarily by modulating the secretion of PRL and LH. Research on Yangzhou geese had revealed the molecular mechanisms of pituitary responses to photoperiods at the transcriptomic level. RNA-seq analysis showed that under different photoperiod treatments, the expression level of the *PRL* gene in pituitary tissue was the highest among all differentially expressed genes, and its dynamic expression pattern was highly consistent with the reproductive state [29]. Furthermore, the “neuroactive ligand-receptor interaction” pathway was significantly enriched in the transcriptomic data, which involved the signal transduction of various neurotransmitters and neuropeptide receptors [30]. This indicated that the pituitary was not merely a terminal for hormone secretion but also a crucial hub integrating multiple environmental and neural signals. Photoperiod signals may regulate the pituitary’s response to various neuroactive substances by affecting higher centers such as the hypothalamus, ultimately achieving fine-tuned regulation of the synthesis and secretion of reproductive hormones like PRL. Collectively, these studies constructed a complete regulatory chain of “photoperiod → neuroendocrine network → dynamic *PRL* expression → reproductive state transition,” providing a solid background and theoretical framework for understanding the expression of PRL-S under specific photoperiods.

Integrating the unique molecular structural characteristics of PRL-S, its specific expression pattern in follicles under short photoperiods, and the precise regulatory background of the photoperiod and *VIP-PRL* axis on avian reproduction, we hypothesized that PRL-S may be an adaptive regulatory factor evolved in Zhedong White Geese in response to short photoperiods. It served to fine-tune the reproductive cycle and balance egg-laying and broodiness behaviors. First, PRL-S was not associated with the broody state induced by high *PRL* levels, instead, it appeared during short photoperiods and early follicular development stages that tend to maintain or promote egg-laying. This suggested that PRL-S may lack the strong pro-broody function of PRL-L and might even exert opposing or modulatory effects. Second, it may act as a “weak agonist” or “partial agonist,” activating a signaling profile distinct from PRL-L, for example, differing in the strength or duration of *JAK-STAT* pathway activation, or preferentially activating other non-classical pathways, thereby producing differentiated biological effects. Finally, the production of PRL-S may represent a post-transcriptional regulatory response to environmental signals. Photoperiods ultimately affected pituitary gene expression through the neuroendocrine network, which may include the regulation of alternative splicing of the *PRL* gene, resulting in varying ratios of PRL-L and PRL-S. Under short photoperiods, promoting the generation of PRL-S may help maintain a certain level of *PRL* signaling while avoiding the excessive production of strongly pro-broody PRL-L, thus facilitating the extension of the laying period. Therefore, the function of PRL-S may not be a simple binary of “present” or “absent,” but rather a “calibration” or “fine-tuning” of the *PRL* signaling system in specific environments. Through its unique structure, it participated in *PRLR* signal transduction in a differentiated manner, playing a refined regulatory role in the complex network translating environmental signals into physiological responses, thereby helping the organism maximize reproductive benefits. This hypothesis provided a clear theoretical orientation and research framework for subsequent in-depth analyses of the biological significance of PRL-S at multiple levels, including cellular function, signaling pathway preference, and population genetic associations.

In summary, this study systematically revealed the molecular characteristics and potential functions of the novel *PRL* alternative splice variant, PRL-S, in Zhedong White Geese. This finding not only enriched our understanding of the complexity of *PRL* gene alternative splicing but also aligned with the recent research consensus on the precise regulation of avian reproductive rhythms by photoperiods via the *VIP-PRL* axis [31]. Although this study primarily validated the receptor-binding and signaling activation potential of PRL-S using in vitro cell models and recombinant proteins, its exact physiological functions, secretion mechanisms, and regulatory networks in vivo still require further validation through in vivo experiments (e.g., gene knockout, antibody neutralization, or overexpression. Future research should focus on its spatiotemporal expression profiling, cellular functional validation, signaling pathway preferences, and genetic association analyses with reproductive performance to comprehensively elucidate the precise role of PRL-S in the avian reproductive regulatory network and evaluate its application value in poultry genetic improvement.

## 4. Materials and Methods

### 4.1. Ethical Statement

All animal experiments in this study were approved by the Institutional Animal Care and Use Committee of Northeast Agricultural University (Protocol code: SRM-08; date of approval: 1 January 2025) to ensure compliance with international animal welfare guidelines.

### 4.2. Sample Collection

The experimental geese were 400-day-old female Zhedong White Geese sourced from Xiangshan Wenjie White Goose Breeding Co., Ltd. in Ningbo, Zhejiang Province, China. According to feeding standard, the geese were raised under a semi-housed and semi-grazing system with ad libitum access to feed and water. The average environment temperature was maintained at 15 ± 2 °C under nature lighting. Samples were collected during the natural breeding season (the short-daylight season, from 5 to 8 November 2025) of Zhedong White Geese. A total of 20 geese were sampled, with 10 collected during the egg-laying period and the remaining 10 during the brooding period. Laying-phase geese were defined as females that laid eggs continuously and exhibited no broody behavior. Broody-phase geese were defined as females that had ceased egg production and displayed intense broody behavior (such as prolonged nest sitting, increased aggressiveness, and puffed-up feathers) for more than 3 days. Geese were first anesthetized via isoflurane inhalation, followed by euthanasia via intravenous injection of sodium pentobarbital after loss of consciousness. The hypothalamus, pituitary, and ovarian tissues were collected. At the same time, follicles at different developmental stages were also collected. SWF: 3–8 mm in diameter. LWF: 8–15 mm in diameter. SYF: approximately 8–15 mm in diameter, with yolk deposition but pale in color. LYF: 15–35 mm in diameter, distinctly golden yellow in color. F1 follicles: 49.34 ± 2.09 mm in diameter; F2 follicles: 43.66 ± 2.23 mm in diameter; F3 follicles: 36.65 ± 3.09 mm in diameter; F4 follicles: 29.50 ± 2.79 mm in diameter; F5 follicles: 19.69 ± 3.65 mm in diameter. Ten follicles at various developmental stages were collected from each goose and pooled into a single sample for analysis. All samples were immediately snap-frozen in liquid nitrogen for subsequent analysis. The samples used for transcriptome sequencing and subsequent validation were all collected from Zhedong White Geese during both the laying period and the broody period, with 10 individuals per period (n = 10). The cDNA used for PCR validation was synthesized via reverse transcription from pooled tissue RNA of these 10 geese to ensure that the detected splice variants were representative of the population.

### 4.3. Transcriptome Sequencing

The experimental samples were sent to Personal Biotechnology Co., Ltd. (Shanghai, China) for transcriptome sequencing. Briefly, total RNA was extracted from pituitary tissue samples of each group. RNA integrity and purity were evaluated using an Agilent 2100 Bioanalyzer (Agilent Technologies, Santa Clara, CA, USA). Only samples with an RNA Integrity Number greater than 7.0 were selected for subsequent library construction. mRNA enrichment and sequencing library preparation were performed using the NEBNext^®^ Ultra^TM^ RNA Library Prep Kit for Illumina^®^ (New England Biolabs, Ipswich, MA, USA). Briefly, eukaryotic mRNA was enriched from total RNA using oligo (dT) magnetic beads. The constructed libraries were sequenced on an Illumina NovaSeq 6000 platform with 150 bp paired-end mode. Approximately 6 Gb of high-quality clean data was generated for each sample. The raw reads were filtered to remove adapter-containing and low-quality sequences to obtain clean reads. Subsequently, the clean reads were aligned to the goose reference genome (NCBI GCF_040182565.1) using HISAT2 software (v2.0.5). The alignment results were used for downstream analyses, and transcript assembly and quantification were performed using StringTie software 3.0.3. The sequencing data underwent rigorous quality control, with all Q30 values exceeding 90%.

### 4.4. RNA Extraction and cDNA Synthesis

Samples were placed in a mortar, ground into a fine powder in liquid nitrogen, and then mixed with 1 mL of RNAiso Plus reagent (TaKaRa, Dalian, China, Cat# 9109). Total RNA was extracted according to the manufacturer’s instructions of the RNA extraction kit. A 0.5-μL aliquot was used to determine RNA concentration and purity. RNA purity was assessed using a NanoDrop 2000 spectrophotometer (Thermo Fisher Scientific, Wilmington, DE, USA), with A260/A280 ratios ranging from 1.8 to 2.1 and A260/A230 ratios greater than 2.0. RNA integrity was evaluated by 1.5% agarose gel electrophoresis, which showed distinct 28S and 18S rRNA bands, with the 28S band exhibiting approximately twice the intensity of the 18S band. Only RNA samples meeting these purity and integrity criteria were used for subsequent experiments. cDNA was synthesized using PrimeScript^TM^ RT reagent Kit (TaKaRa, Dalian, China, Cat# RR047A) following the manufacturer’s protocol [32].

### 4.5. Identification of PRL Alternative Splice Variants

Samples were collected from 10 geese in the laying period and 10 geese in the broodiness period of Zhedong White geese. Based on the splice variant sequences, primers targeting the full-length CDS region were designed using SnapGene software 8.2.2 and synthesized by Heilongjiang Jiansu Gene Technology Co., Ltd. (Harbin, China) (Table 3). PCR amplification was performed using cDNA as the template. The reaction system consisted of 5 μL of 2× Taq Master Mix, 0.5 μL of cDNA, 0.4 μL of each forward and reverse primer (10 μmol/L), and 3.7 μL of ddH_2_O. The thermal cycling conditions were as follows: initial denaturation at 95 °C for 2 min; 35 cycles of 95 °C for 30 s, 52 °C for 30 s, and 72 °C for 30 s; and a final extension at 72 °C for 2 min, followed by storage at 4 °C. The PCR products were analyzed via 2% agarose gel electrophoresis and recovered using a gel extraction kit according to the manufacturer’s instructions. The recovered products were ligated into the pMD19-T vector using a TA cloning kit and transformed into DH5α competent cells. The transformed cell suspension was plated onto LB agar plates containing 100 μg/mL ampicillin and incubated at 37 °C for 24 h. Single colonies were picked and inoculated into 1 mL of LB liquid medium containing 100 μg/mL ampicillin, then cultured overnight at 37 °C with shaking. Colony PCR using M13 primers was performed to verify the TA cloning. The colony PCR system consisted of 5 μL of 2× Taq Master Mix, 0.5 μL of bacterial suspension, 0.4 μL of each forward and reverse primer (10 μmol/L), and 3.7 μL of ddH_2_O. The amplification products were verified by 2% agarose gel electrophoresis. Ten individual colonies were picked separately for colony PCR verification. A 200 μL aliquot of the bacterial culture that met the amplification criteria was sent to a commercial sequencing company for plasmid extraction and Sanger sequencing. All sequencing results were completely consistent, confirming the accuracy of the amplified products.

### 4.6. Bioinformatics Analysis

The amino acid sequences of the *PRL* alternative splice variants were deduced using ExPASy Translate tool. The mRNA and amino acid sequences were aligned using DNAMAN 9.0. Physicochemical properties and protein structures were predicted and analyzed using online software tools (Table 4). Protein hydrophilicity/hydrophobicity analysis was performed using the Kyte-Doolittle algorithm. The target protein sequences were scanned using the ProtScale tool. To balance curve smoothing and the identification of local features, the sliding window size was set to 9 amino acid residues. Hydrophobicity scores were calculated based on the Kyte-Doolittle scale, where positive values indicate hydrophobicity and negative values indicate hydrophilicity.

### 4.7. Construction and Transformation of PRL Recombinant Expression Plasmids

The recombinant expression plasmids for PRL-L and PRL-S were constructed using restriction enzyme digestion and ligation. PCR amplification was performed using templates containing the complete coding sequences of PRL-L and PRL-S, respectively. Specific primers were designed based on the multiple cloning site of the pET-28a vector, introducing NcoI and XhoI restriction sites at the 5′ and 3′ ends, respectively. The reading frame was strictly verified during primer design to ensure that the inserted fragments were in-frame with the N-terminal 6× His tag sequence of the vector. After purification, the PCR products and the empty pET-28a vector were digested with NcoI and XhoI restriction enzymes. The digested products were purified from an agarose gel and ligated using T4 DNA ligase overnight at 16 °C. The ligation products were then transformed into *E. coli* DH5α competent cells and plated on LB agar containing kanamycin to screen for positive clones. Single colonies were picked, cultured, and plasmids were extracted and sent for sequencing verification.

### 4.8. Expression and Analysis of Recombinant PRL Proteins

Single colonies were picked and cultured overnight in LB liquid medium containing kanamycin. The cultures were then inoculated at a 1:100 dilution into fresh kanamycin-containing LB liquid medium and incubated at 37 °C until the OD600 reached 0.6–1.0. Isopropyl β-D-1-thiogalactopyranoside (IPTG, 1 mol/L, Sigma-Aldrich, St. Louis, MO, USA) was added to a final concentration of 0.5 mmol/L, and the cultures were incubated for an additional 4 h. The bacterial cells were harvested and resuspended in 1× PBS buffer at a ratio of cell mass (g) to buffer volume (mL) of 1:10. Phenylmethylsulfonyl fluoride (PMSF), lysozyme, DNase I, and RNase A were added to final concentrations of 1 mmol/L, 0.4 mg/mL, 10 μg/mL, and 20 μg/mL, respectively. The cell suspensions were frozen at −80 °C for 5 min until solidified, then thawed at room temperature. This freeze–thaw cycle was repeated five times. The bacterial lysates were centrifuged at 19,320× *g* for 15 min at 4 °C to separate the supernatant from the pellet, with the pellet representing inclusion bodies. The pellet was resuspended in 1× SDS Loading Buffer to match the original volume of the bacterial lysate, while the supernatant was mixed with a 1/4 volume of 5× SDS Loading Buffer. Both samples were heat-denatured at 100 °C for 10 min. The expression of the recombinant proteins was analyzed via SDS-PAGE with a 10 μL sample load.

### 4.9. Purification of Recombinant PRL Proteins

Inclusion bodies obtained from 50 mL of bacterial culture were dissolved by adding 5 mL of Binding Buffer (8 mol/L urea, 50 mmol/L NaH_2_PO_4_, 5 mmol/L imidazole, 100 mmol/L Tris-HCl, pH 8.0) and shaking at 4 °C for 30 min. Following centrifugation, the supernatant (denatured protein solution) was separated from the pellet. The supernatant was transferred to His-Tag magnetic beads (Beijing Solarbio Science & Technology Co., Ltd., Beijing, China) pre-equilibrated with Binding Buffer and incubated with shaking at 4 °C for 1 h. The volume of magnetic beads used in each purification experiment was 100 μL, with a binding capacity of approximately 80 μg for His-tagged proteins. The magnetic beads were separated using a magnetic rack, and the flow-through was collected. To remove non-specifically bound proteins, the beads were washed three times with 10 mL of Washing Buffer (8 mol/L urea, 50 mmol/L NaH_2_PO_4_, 20 mmol/L imidazole, 100 mmol/L Tris-HCl, pH 8.0), and the wash fractions were retained. The target protein was eluted using 2 mL of Elution Buffer (8 mol/L urea, 50 mmol/L NaH_2_PO_4_, 500 mmol/L imidazole, 100 mmol/L Tris-HCl, pH 8.0). The elution step was repeated three times, and the eluates were collected. For the subsequent refolding experiments, the two elution fractions with the highest protein concentrations were pooled. The purification efficiency was evaluated via SDS-PAGE.

### 4.10. Refolding of Purified Recombinant PRL Proteins and Co-Immunoprecipitation

The collected eluates were transferred into dialysis tubing and dialyzed against 100 volumes of 1× PBS (pH 7.2–7.4) for 24 h. The contents of the dialysis bag were then transferred to a centrifuge tube and centrifuged at 19,320× *g* for 15 min at 4 °C. The supernatant was discarded, and the pellet was resuspended in refolding buffer and incubated at 4 °C for 1 h. After centrifugation, the supernatant was collected. His-Tag magnetic beads were used to assess the burial of the 6× His tag. The supernatant was transferred back into dialysis tubing and dialyzed for an additional three days. Following centrifugation, the final supernatant containing the refolded recombinant protein was collected and stored at −80 °C. The refolding efficiency was verified via SDS-PAGE, and the protein concentration was determined using a BCA Protein Assay Kit. For the co-immunoprecipitation (CO-IP) assay, 0.2 g of follicle tissue from Zhedong White Geese was homogenized on ice in 400 μL of CO-IP cell lysis buffer supplemented with a protease inhibitor cocktail using a tissue homogenizer. The homogenate was transferred to a centrifuge tube and agitated by inversion at 4 °C for 2 h. Following centrifugation, the supernatant was collected as the tissue lysate. A total of 200 μL aliquot of the recombinant PRL protein solution was mixed with 200 μL of the tissue lysate and incubated with inversion at 4 °C for 2 h. Rabbit IgG and *PRLR* antibodies were then added to a final concentration of 50 μg/mL, and the mixture was incubated overnight at 4 °C with inversion. Subsequently, 40 μL of pre-washed Protein A + G magnetic beads were added, and the mixture was incubated at room temperature with inversion for 1 h. The beads were separated using a magnetic rack, and the supernatant was discarded. The beads were washed three times with 1× TBST. Finally, 100 μL of 1× SDS Loading Buffer was added to the beads, and the samples were heat-denatured at 95 °C for 5 min. The protein–protein interactions were detected via Western Blotting using anti-His-tag and anti-*PRLR* antibodies.

### 4.11. Construction and Validation of the STAT5 Luciferase Reporter Assay System

Based on the *PRLR* gene sequence, primers were designed to amplify the full-length CDS sequence, with *Hind*III and *Xho*I restriction sites incorporated at the 5′ ends of the forward and reverse primers, respectively. The PCR products were cloned into the pcDNA3.1 vector for sequencing. Five tandem *STAT5* binding sequences were synthesized and directionally cloned into the *Hind*III and *Xho*I sites of the pGL3-BASIC vector to construct the recombinant plasmid pGL3-5× (*STAT5*). HEK293T cells cultured in 96-well plates were co-transfected with 100 ng of pcDNA3.1-*PRLR* plasmid, 100 ng of pGL3-5× (*STAT5*) plasmid, and 2 ng of pRL-TK plasmid for 6 h using Lipofectamine 3000 reagent (Thermo Fisher Scientific, Waltham, MA, USA). The pGL3-Control plasmid was used in place of the pGL3-5× (*STAT5*) plasmid as a positive control. The treatment group was cultured for 24 h in DMEM supplemented with 60 nM (Concentration screened in the preliminary experiment) recombinant *PRL* and 10% fetal bovine serum (FBS), whereas both the negative and positive control groups were cultured in DMEM supplemented with 10% FBS only. Each sample was repeated three times. Cells were processed using a Dual-Luciferase Reporter Assay System (Promega, Madison, WI, USA). The entire experiment (from cell plating and transfection to detection) was independently repeated three times. In each independent experiment, each treatment group was set up in triplicate, and the final results were calculated as the mean values. The relative luciferase activity was measured using a Multilabel Plate Reader (Thermo Fisher Scientific, Waltham, MA, USA).

### 4.12. Relative mRNA Expression Analysis

Total RNA was extracted using TRIzol reagent (Thermo Fisher, Waltham, MA, USA) according to the manufacturer’s instructions. cDNA was synthesized using the PrimeScript^TM^ RT Kit (TaKaRa, Dalian, China, Cat# RR047A). The primer sequences were listed in Table 3. Specific primers were synthesized by Heilongjiang Jiansu Technology Co., Ltd. (Heilongjiang, China). Detailed procedures had been described in a previous publication [33]. Briefly, Spectrophotometer (Healthcare BioSciences AB, Uppsala, Sweden) was used to determine RNA purity. OD260/OD280 was between 1.8 and 2.1. cDNA was synthesized using PrimeScript^TM^ RT reagent Kit (TaKaRa, Otsu, Japan) in a volume of 60 μL (containing 5 μg of the total RNA) according to the manufacturer’s instructions. Synthesized cDNA was diluted fivefold with sterile water and was stored at − 20 °C until the next step. Quantitative real-time PCR was performed using LightCycler^®^ 96 (Roche, Life Science, Basel, Basel-Stadt, Switzerland) with the SYBR^®^ PrimeScript^TM^ RT-PCR Kit (Roche, Basel, Switzerland) according to the manufacturer’s instructions. Reactions were performed in a 10-μL reaction mixture containing 5 μL of the 2× SYBR Green I PCR Master Mix, 1 μL cDNA, 0.3 μL of each primer (10 μM), and 3.4 μL of PCR grade water. PCR procedure consisted of 95 °C for 10 min, followed by 40 cycles of denaturing-annealing/elongating (95 °C for 15 s and 60 °C for 1 min), and melting curve analysis (95 °C for 15 s and 60 °C for 20 s). The melting curve analysis showed only one peak for each PCR product. Each sample was repeated three times. Relative mRNA expressions were calculated according to the 2^−ΔΔCT^ method. GAPDH was selected as the reference gene [34,35].

### 4.13. Western Blot Analysis

A total of 0.1 g sample was placed into a beaker, to which 1 mL of RIPA lysis buffer (Seven, Beijing, China) and 50 μL of PMSF (Biosharp, Beijing, China) were added. The final working concentration of PMSF was 5 mmol/L. The sample was fully lysed using a tissue homogenizer (HT Homogenizer^TM^, Beijing, China). Following centrifugation, the supernatant was collected, and the protein concentration was determined using a BCA Protein Assay Kit (Beyotime Biotechnology, Shanghai, China). Proteins were separated by 10% SDS-PAGE at 200 mA for 2 h and transferred onto PVDF membranes (Seven, Beijing, China). For *PRL* detection, a rabbit polyclonal antibody (Affinity, Changzhou, Jiangsu, China, Cat# DF6506) was used. The membranes were blocked with 5% skim milk for 2 h at room temperature and then incubated with primary antibodies at 4 °C overnight. The membrane was washed three times with TBST buffer, 10 min each time. After washing with TBST (Seven, Beijing, China), the membranes were incubated with an anti-rabbit secondary antibody (Immunoway, Beijing, China) at room temperature for 2 h. The membranes were washed again and imaged using a molecular imaging system. The primary antibodies recognized the C-terminal epitopes, allowing the detection of both isoforms, and the separation was based on molecular weight differences. The primary antibodies (Affinity, Changzhou, Jiangsu, China) were used at dilutions of 1:2000 for PRL-S and PRL-L, and 1:3000 for GAPDH. The anti-rabbit IgG secondary antibody (Immunoway, Beijing, China) was used at a dilution of 1:1000. Protein bands were visualized using a Bio-Rad molecular imaging system (Bio-Rad, Hercules, CA, USA). Each sample was repeated three times. Detailed procedures had been described in a previous publication [36].

### 4.14. Statistical Analysis

Experimental data were expressed as mean ± standard deviation and were analyzed using SPSS 31.0 statistical software. Before analysis, the normality of residuals and homogeneity of variances were checked using the Shapiro–Wilk test and Levene’s test, respectively. For tissue expression data, a three-way ANOVA was used to evaluate the main effects of physiological state, tissue type, and *PRL* variant, as well as their interactions. For follicular expression data, a two-way ANOVA was used. When a significant F-test was obtained, Duncan’s multiple range test was used for post hoc pairwise comparisons. The false discovery rate for all qPCR and Western blot data was controlled using the Benjamini–Hochberg method. Differences were considered statistically significant at *p* < 0.05. Samples from all 20 geese were included in the final analysis.

## 5. Conclusions

In this study, we successfully identified a novel alternatively spliced variant of the *PRL* gene in Zhedong White geese, designated as PRL-S. Although PRL-S exhibited significant structural differences from the full-length PRL-L, in vitro experiments demonstrated that the recombinant PRL-S protein can specifically bind to the *PRLR* and effectively activate the downstream *JAK*-*STAT5* signaling pathway. Expression profile analysis revealed that PRL-S was expressed in the hypothalamic–pituitary–gonadal axis and follicles, and its expression level was closely associated with specific tissue types and reproductive stages. These findings suggest that PRL-S may be an active molecule with potential physiological functions. However, given that this study was primarily based on in vitro experiments and expression correlation analyses, the exact physiological functions of PRL-S in vivo, as well as its specific regulatory mechanisms underlying broodiness and egg-laying performance, remained to be validated through further in vivo gain- or loss-of-function experiments.

## Figures and Tables

**Table 1:** Analysis of the physicochemical properties of *PRL* alternative splice variant proteins.

Name	PRL-S	PRL-L
Molecular weight	16,540.14	22,782.82
Amino acid length	142	199
Isoelectric point	6.65	5.66
Instability index	58.24	50.01
Aliphatic index	113.24	93.53
Grand average of hydropathy	−0.257	−0.378
Abs 280 nm (M^−1^ cm^−1^)	1.268–1.283	1.051–1.068

**Table 2:** Analysis of the secondary structure composition of *PRL* alternative splice variant proteins (%).

Name	α-Helix	Extended Strand	β-Turn	Random Coil
PRL-S	67.61	1.41	2.11	28.87
PRL-L	61.81	1.01	2.51	34.67

**Table 3:** Primers used in this study.

Gene	Primer Sequence	Size (bp)
PRL-S	Forward 5′-TGAAGACAGTCGTCTTCAGCCT-3′ Reverse 5′-TTAGCAATTGCTATCATGTATTAGGCGGC-3′	429
PRL-L	Forward 5′-ATGAGCACCAAGGGGGCTT-3′ Reverse 5′-TTAGCAATTGCTATCATGTATTAGGCGGC-3′	690
GAPDH	Forward 5′-AGAACATCATCCCAGCGT-3′ Reverse 5′-AGCCTTCACTACCCTCTTG-3′	128

**Table 4:** Bioinformatics online analysis software.

Software	Access Date	Website	Function	Parameters
ProtParam	18 May 2025	https://web.expasy.org/protparam	Physicochemical properties analysis of proteins	Default parameters were used to analyze physicochemical properties.
Protscale	12 March 2026	https://web.expasy.org/protscale/	Hydrophilicity and hydrophobicity analysis of proteins	Kyte-Doolittle scale; window size was set to 9 amino acid residues.
SignalP 5.0	12 March 2026	https://services.healthtech.dtu.dk/service.php?SignalP-5.0	Signal peptide analysis	Default parameters for eukaryotes were used for signal peptide prediction.
SOPMA	12 June 2025	http://npsa-prabi.ibcp.fr/cgi-bin/npsa_automat.pl?page=/NPSA/npsa_sopma.html	Protein secondary structure analysis	Default parameters were used for secondary structure prediction.
Swiss-Model	28 March 2026	https://www.swissmodel.expasy.org/	Protein tertiary structure analysis	The server automatically selected

## Data Availability

The original contributions presented in this study are included in the article. Further inquiries can be directed to the corresponding authors.
